# Standard: human liver-on-a-chip

**DOI:** 10.1186/s13619-025-00226-0

**Published:** 2025-03-24

**Authors:** Haitao Liu, Xu Zhang, Yaqing Wang, Min Zhang, Peng Wang, Jing Shang, Zhongqiang Li, Likun Gong, Xin Xie, Dongyang Liu, Jingbo Pi, Xinghua Gao, Xianliang Li, Wei Ding, Dianbing Wang, Yun Long, Lan Wang, Song Li, Xingchao Geng, Pingkun Zhou, Wanjin Tang, Xian’en Zhang, Chunying Chen, Shengli Yang, Jianhua Qin

**Affiliations:** 1 Division of Biotechnology, Dalian Institute of Chemical Physics, Chinese Academy of Sciences, Dalian, China; 2 University of Science and Technology of China, Hefei, China; 3 Suzhou Institute for Advanced Research, University of Science and Technology of China, Suzhou, China; 4 China Pharmaceutical University, Nanjing, China; 5 China National Institute of Standardization, Beijing, China; 6 Shanghai Institute of Materia Medica, Chinese Academy of Sciences, Shanghai, China; 7 Peking University Third Hospital, Beijing, China; 8 China Medical University, Shenyang, China; 9 Shanghai University, Shanghai, China; 10 Department of HBP Surgery, Beijing Chao Yang Hospital, the Capital Medical University, Beijing, China; 11 SPH KDL Health Beijing, Shanghai Pharma, Beijing, China; 12 Institute of Biophysics, Chinese Academy of Sciences, Beijing, China; 13 Peking Union Medical College Hospital, Beijing, China; 14 Dalian Xin’en Medical Technology Co., LTD, Dalian, China; 15 Institute of Pharmacology and Toxicology, Academy of Military Medical Science, Beijing, China; 16 National Institutes for Food and Drug Control, Beijing, China; 17 Institute of Radiation Medicine, Chinese Academy of Medical Sciences, Tianjin, China; 18 Shenzhen University of Advanced Technology, Shenzhen, China; 19 National Center for Nanoscience and Technology, Beijing, China; 20 Beijing Institute for Stem Cell and Regenerative Medicine, Beijing, China

**Keywords:** Organs-on-chips, Liver-on-a-chip, Microphysiological system, Standard

## Abstract

Organs-on-chips are microphysiological systems designed to replicate key functions of human organs, thereby accelerating innovation in life sciences, such as disease modeling, drug development, and precision medicine. However, the lack of standardized definitions, structural designs, cell sources, model constructions, and functional validations has posed challenges to their widespread translational applications. On April 29, 2024, the Chinese Society of Biotechnology introduced "Organs-on-chips: Liver", China's first group standard for human liver-on-a-chip technology. This pioneering standard provides comprehensive guidelines, including scope, terminology, definitions, technical requirements, detection methods, and quality control measures for developing liver models on chips. The introduction of this standard is set to facilitate the establishment of institutional protocols, promote widespread adoption, and drive the international standardization of liver-on-a-chip technologies.

**Supplementary Information:**

The online version contains supplementary material available at 10.1186/s13619-025-00226-0.

## Scope

This document specifies the ethical requirements, technical requirements, and detection methods for construction and evaluation of human liver-on-a-chip.

This document is applicable to the construction, detection and evaluation activities of functional liver-on-a-chip.

## Normative references

The contents of the following documents are essential provisions of this document through normative reference. For referenced documents with specified dates, only the version corresponding to that date is applicable to this document; for documents without specified dates, the latest version (including all amendments) is applicable to this document:

GB/T 38736–2020 Ethical Requirements of Human Biobanking.

GB/T 16886.5–2017 Biological evaluation of medical devices-Part 5: Tests for in vitro cytotoxicity.

GB/T 42466–2023 Technical Specification for Pluripotent Stem Cells Management of Biobanking.

WS 213 Diagnostic for Hepatitis C.

WS 293 Diagnostic for HIV/AIDS.

WS 299 Diagnostic Criteria for Viral Hepatitis B.

T/CSB 0003–2024 General Terminology of Organs-on-chips.

T/CSCB 0001–2020 General Requirements for Stem Cells.

T/CRHA 017–2023 Guidelines for the Establishment, Quality Control, and Storage of Human Hepatic Progenitor Cell Derived Organoids.

## Terms and definitions

The following terms and definitions apply to this document:

### Liver-on-a-chip

A hepatic microphysiological system that can reflect the key structures and biofunctions (such as metabolism, synthesis, and secretion) of the human liver by seeding multiple cells (e.g., hepatocytes and nonparenchymal cells) and reconstructing microenvironmental elements (e.g., extracellular matrix and fluids) (T/CSB 0003–2024, 5.7).

### Hepatic microenvironment

The integration of biophysical and biochemical factors in hepatic tissue that affects cell growth and functions, including various cell types, extracellular matrix, fluid stimuli and physicochemical gradients (Beckwitt 2018).

### Hepatocyte

The predominant cell population in liver tissue that plays crucial roles in bile secretion, synthesis of diverse plasma proteins, and pivotal functions, including carbohydrate metabolism, lipid metabolism, and the metabolism of hormones and drugs (Human Anatomy Nomenclature Committee Approval, 2014).

### Hepatic nonparenchymal cells

The cells in liver that do not constitute the functional units but play supportive and regulatory roles, mainly including hepatic sinusoidal endothelial cells, Kupffer cells, and hepatic stellate cells.

### Organoid

Self-assembled 3D microtissues derived from stem cells or organ-specific precursor cells through self-renewal, differentiation, and self-organization, which consist of multiple cell types and can partially reflect the key structures and specific functions of their source tissue or organ (T/CSB 0003–2024, 3.3).

### Liver organoid

Self-assembled 3D microtissues derived from induced pluripotent stem cells, embryonic stem cells or liver progenitor cells through self-renewal, differentiation, and self-organization, which consist of multiple hepatic cell types and can partially reflect the key structures and specific functions of in vivo liver tissue (Wang 2018b; Wang 2020).

### Intrinsic clearance

The ratio of the drug elimination rate to the concentration of unbound drug at the enzyme site, which is calculated based on the plasma concentration of the unbound drug and serves as an objective measure of the liver's ability to clear drugs, and unaffected by plasma protein binding (Wang 2005).

## Ethical requirements

### General principles

When the preparation and evaluation process of a liver-on-a-chip involves the use of human primary tissues or cells, which raises ethical concerns, ethical approval should be obtained, and informed consent should be obtained to ensure the privacy protection of the donors.

### Informed consent

Prior to the collection of human samples, written informed consent should be obtained from the donors, clearly outlining the rights and responsibilities of both the donors and the sample collection entity. Informed consent should comply with the requirements of Section. 5.3 of GB/T 38736–2020.

### Ethical review

The establishment and research protocols of the liver-on-a-chip should be reviewed and approved by the ethics review committee of the project's sponsoring and primary executing institution.

### Privacy protection

Human sample donors have the right to personal privacy, and the confidentiality and protection of their personal privacy information should comply with the requirements of Chapter 6 of GB/T 38736–2020.

## Construction of liver-on-a-chip

### Technical requirements

#### Fabrication of the chip

##### Design of the Chip

Liver-on-a-chip typically comes in two forms of tissue-tissue interface and 3D culture (Lee 2007; Lee 2013; Bhise 2016).

Liver-on-a-chip with tissue-tissue interface is composed of two independent chambers that are separated by porous membranes, used for the culture of hepatocytes and hepatic nonparenchymal cells, respectively.

Note: Fluid shear stress is usually applied on the nonparenchymal cell side for dynamic cell culture in liver-on-a-chip.

Liver-on-a-chip for 3D culture typically encapsulate hepatocytes and hepatic nonparenchymal cells in matrix to form cell spheroids.

##### Material selection for the chip

The commonly used material for the liver-on-a-chip is PDMS (Li 2018; Yin 2021; Tao 2022).

Other materials for the liver-on-a-chip typically include PMMA, PS, and PC.

For liver-on-a-chip with tissue-tissue interface, the material of the porous membrane can be selected from PDMS, PC, PET, and nylon, with pore sizes ranging from 0.01 μm to 10 μm.

##### Methods for the chip fabrication

PDMS chips are commonly fabricated using soft lithography methods.

Chips made of materials such as PMMA, PS, and PC are preferably manufactured using techniques such as injection molding and 3D printing.

For PDMS chips, plasma bonding is typically used for processing; for plastic materials such as PMMA, PS, and PC, methods such as heat sealing, ultrasonic sealing, and adhesive sealing are commonly employed.

##### Quality control for the chip

The cytotoxicity of chips should be tested using methods outlined in GB/T 16886.5–2017. The chips should meet the non-cytotoxicity requirements outlined in Chapter 8 of GB/T 16886.5–2017.

#### Seeding cells in the chip

##### Cell components

Liver-on-a-chip should contain human hepatocytes.

Note: The sources of hepatocytes typically include hepatic cell lines (such as HepaRG, HepG2), primary human hepatocytes, stem cells or liver organoids.

When used to simulate complex physiological functions of the liver (such as immunologic process, liver regeneration, and liver metabolism) and pathological processes (such as liver fibrosis and cirrhosis), liver-on-a-chip should include endothelial cells.

Note: Endothelial cells in liver-on-a-chip should typically be derived from primary human umbilical vein endothelial cells, primary human liver sinusoidal endothelial cells, or differentiated endothelial cells from stem cells (such as induced pluripotent stem cells and embryonic stem cells), with a maximum passage number of 10.

The types of hepatic nonparenchymal cells used in liver-on-a-chip should be adjusted according to different usage requirements:

- When simulating the immune function in the liver microenvironment, liver-on-a-chip should include immune cells such as Kupffer cells.
- When conducting research on liver fibrosis, both hepatic stellate cells and Kupffer cells should be used in liver-on-a-chip.

##### Requirements of cell performance

The typical requirements for cell performance in the chip include:

- Cell Morphology
  All the cell types should exhibit typically cellular morphologies before seeding, which can be referenced from the ATCC official website.
  HepaRG and HepG2 cells typically appear as oval tubular or spindle-shaped and tend to grow in clusters.
  Liver organoids should appear as cystic or bud-like structures under an optical microscope, with a central cavity surrounded by closely apposed polygonal epithelial cells. The edge of the cavity where it meets the outside should be clear, and the cells should be translucent.
  Endothelial cells should appear as flat polygons or spindle-shaped.
  Kupffer cells have irregular shapes and often attach to other cells' surfaces as plate-like or filamentous pseudopodia.
  Hepatic stellate cells typically appear star-shaped, spindle-shaped, polygonal, or spindle-shaped with pseudopodia.
- Chromosomal Karyotype
  The normal chromosomal karyotype of cells should be 46, XY or 46, XX.
- Microbiological Testing
  Microbiological detection indicators include:
  i) Bacteria and Fungi
    Testing should follow the "Sterility Test" method outlined in the "Pharmacopoeia of the People's Republic of China (2020 Edition, Part III)". After culturing by membrane filtration or direct inoculation for no less than 14 days, observe whether the culture medium becomes turbid, or take culture fluid smears, stain, and microscopically examine (The Pharmacopoeia Committee, 2020).
  ii) Chlamydia
    Testing should follow the "Chlamydia Test" method outlined in the "Pharmacopoeia of the People's Republic of China (2020 Edition, Part III)". Testing can be done using Chlamydia culture method or indicator cell culture method (DNA staining), or other methods approved by the national drug regulatory authority (The Pharmacopoeia Committee, 2020).
  iii) HIV
    Testing should be conducted using the WS 293 method, with the concentration of HIV-related RNA below the detection limit in routine PCR.
  iv) HBV
    Testing should be conducted using the WS 299 nucleic acid method, with HBV-related RNA below the detection limit in routine PCR.
  v) HCV
    Testing should be conducted using the WS 213 nucleic acid method, with HCV-related RNA below the detection limit in routine PCR.
  vi) Exogenous Viral Factors
    Testing should follow the "Exogenous Viral Factor Test" method outlined in the "Pharmacopoeia of the People's Republic of China (2020 Edition, Part III)", using methods such as cell culture, inoculation of suckling mice or chicken embryos, nucleic acid amplification technology, etc (The Pharmacopoeia Committee, 2020).
  vii) STR
    Extracted primary cells should undergo STR testing and typing consistent with the donor STR, and microbial testing should yield negative results.

When using stem cells in liver-on-a-chip, compliance with Chapter 5 and Chapter 6 of T/CSCB 0001–2020, and Chapter 8 of GB/T 42466–2023 is required.

When using liver organoids in liver-on-a-chip, the expression and function of organoid cellular biomarkers should comply with the requirements of Sections. 7.6 and 7.7 of T/CRHA 017–2023.

#### Dynamic culture in the chip

##### Perfusion in the chip

Injection pumps, peristaltic pumps, or gravity can be used to drive fluid flow in chips to simulate the dynamic flow microenvironment for cell culture.

##### Range of the shear stress

The range of shear forces produced by the fluid in the chip culture medium is typically between 0.001 dyn/cm^2^ to 5 dyn/cm^2^.

### Construction methods

#### Method for the chip fabrication

The preparation method of the liver chip carrier should comply with the provisions of Supplementary 1: Appendix A.

#### Method for seeding cells in the chip

The method of cell seeding in liver chip should comply with the provisions of Supplementary 1: Appendix B, sections B.3 and B.4.

#### Method for culturing cells in the chip

The cell culture method of liver chip should comply with the provisions of Supplementary 1: Appendix B, section B.5. The method for calculating fluid shear force in liver chips should comply with the provisions of Supplementary 1: Appendix C.

## Validation of liver-on-a-chip

### Requirements of function testing

#### Liver tissue morphology

##### Hepatocytes

In liver tissue, hepatocytes are densely arranged, irregularly wedge-shaped; cells are polygonal, with large and round nuclei, and some cells have binucleation.

##### Hepatic nonparenchymal cells

In liver-on-a-chip, endothelial cells can form a tightly arranged cell layer with stellate cells, wherein:

- Stellate cells are polygonal with pseudopodia;
- Endothelial cells are diamond-shaped or polygonal.

#### Liver biomarker expression

##### Immunofluorescence characterization

In the liver-on-a-chip, the following markers can be detected (Ewart 2022):

- Mature hepatocyte markers: CYP3A4, ALB, and HNF4A, etc.;
- Endothelial cell markers: VWF and VE-Cadherin, etc.;
- Stellate cell markers: Vimentin, etc.

##### H&E characterization

A side view can observe the multi-cellular structural liver tissue barrier.

#### Albumin secretion

The average albumin secretion of liver cells cultured in liver chips for 24 h should reach: 100 ng/(10^6^ cells·24 h) or above.

#### Function of drug metabolism enzyme

The intrinsic clearance rate of cells in liver chips should reach: 10 μL/(10^6^ cells·24 h) or above (Sung 2010; Esch 2015).

### Validation methods for the chip biofunctions

#### Observation of tissue morphology

##### Hepatocytes

During the cell culture process in liver-on-a-chip, daily observations should be conducted under inverted phase-contrast microscopy to track changes in hepatocyte morphology.

##### Hepatic nonparenchymal cells

During the cell culture process in liver-on-a-chip, daily observations should be conducted under inverted phase-contrast microscopy to track changes in nonparenchymal cells morphology.

#### Detection of liver biomarker expression

##### Immunofluorescence characterization

The immunofluorescence characterization of cells in liver chips should comply with the methods in Supplementary 1: Appendix D sections D.3.1, D.3.2, and D.3.4.

##### H&E characterization

The H&E characterization of cells in liver chips should comply with the methods in Supplementary 1: Appendix D sections D.3.1, D.3.3, and D.3.4.

#### Assessment of albumin secretion

The detection of albumin secretion in liver chips should comply with the methods in Supplementary 1: Appendix E sections E.3.1 to E.3.4. The data processing process should comply with the method in Supplementary 1: Appendix E section E.3.5.

#### Functional determination of drug metabolism enzyme

The determination of drug metabolism enzyme function in liver chips should comply with the method in Supplementary 1: Appendix F section F.5. The data processing process should comply with the method in Supplementary 1: Appendix F section F.6 (Wang 2018a; Jang 2019).

## Supplementary Information

Supplementary Material 1. Appendix A-F.

## Data Availability

Not applicable.

## Abbreviations

PDMS: Polydimethylsiloxane
PMMA: Polymethyl Methacrylate
PC: Polycarbonate
PS: Polystyrene
PET: Polyethylene Terephthalate
ATCC: American Type Culture Collection
PCR: Polymerase Chain Reaction
CYP450: Cytochrome P450
ALB: Albumin
CYP3A4: Cytochrome P450 3A4 enzyme
HNF4A: Hepatocyte Nuclear Factor 4 Alpha Gene
VWF: Von Willebrand Factor
VE-Cadherin: Vascular Endothelial Cadherin
H&E: Hematoxylin and Eosin staining
CYP1A2: Cytochrome P450 1A2 enzyme
CYP2B6: Cytochrome P450 2B6 enzyme
