# Supplementary material for: Standard: human liver-on-a-chip
Source: Cell Regen. 2025 Mar 24;14:9. doi: 10.1186/s13619-025-00226-0 (PMC11930896; doi:10.1186/s13619-025-00226-0)
Supplement: Supplementary file 1 — Supplementary Material 1. Appendix A-F. [file 13619_2025_226_MOESM1_ESM.docx]

Supplementary Material

**Appendix A**

(Normative Appendix)

Chip Fabrication: Soft Lithography

**A.1 Instruments**

A.1.1 Spin coater.

A.1.2 Hotplate.

A.1.3 Oven.

A.1.4 Ultraviolet (UV) exposure system.

A.1.5 Horizontal shaker.

A.1.6 Vacuum dryer.

A.1.7 Plasma cleaner.

**A.2 Reagents**

A.2.1 Unless otherwise specified, all reagents used are analytical grade, and water is ultrapure water.

A.2.2 Photoresist.

A.2.3 Developer solution (negative resist developer generally contains organic solvents such as xylene and ethyl lactate; positive resist developer is usually a strong alkaline solution, such as potassium hydroxide and tetramethylammonium hydroxide).

A.2.4 PDMS monomer.

A.2.5 PDMS initiator.

A.2.6 Substrate: glass or silicon wafer.

A.2.7 Silanization agent: typically, dimethyldichlorosilane or trimethylchlorosilane.

**A.3 Fabrication Procedures**

A.3.1 Substrate Pre-treatment: Drying

Before fabricating the chip template, ensure to place the substrate (A.2.6) in an oven (A.1.3) for at least 1 hour to eliminate moisture. Set the oven temperature between 150°C to 200°C for this process.

A.3.2 Chip Template Fabrication: Coating and Pre-baking

Place the substrate in the center of the spin coater (A.1.1), add photoresist (A.2.2) to the center, and adjust the rotation speed to achieve the desired thickness. Then, place the substrate on a preheated hotplate (A.1.2) for pre-baking.

A.3.3 Chip Template Fabrication: Exposure and Post-baking

Affix the pre-designed and printed chip mask onto the substrate after pre-baking, and expose it in a UV exposure system (A.1.4). The exposure intensity and time depend on the photoresist thickness. Then, post-bake the substrate on a preheated hotplate after exposure.

A.3.4 Chip Template Fabrication: Development and Hard Baking

Place the chip template, cooled to room temperature, into a suitable container. Add developer solution (A.2.3) to fully cover the substrate, and gently agitate it either on a horizontal shaker (A.1.5) or by continuous agitation with the developer solution. Stop the development process once the microstructures on the substrate are fully revealed and the edges are clear. Dry the template surface using inert gas, such as nitrogen, to remove any residual moisture. Finally, hard bake the template at temperatures ranging from 150°C to 200°C to ensure its stability and durability for subsequent use.

A.3.5 Chip Template Fabrication: Surface Passivation

Place the chip template, cooled to room temperature, into a vacuum dryer (A.1.6). Simultaneously, add a small amount of silanization agent (A.2.7) to the side of the template, allowing the silanization agent's gas to fully deposit on the template surface at room temperature.

A.3.6 Fabrication and Sealing of PDMS Chip

Mix PDMS monomer (A.2.4) and initiator (A.2.5) in a specified volume ratio, typically 10:1. Pour the mixture onto the chip template to achieve the desired thickness. Heat the PDMS-containing chip template in an oven at 60°C to 100°C for at least 30 minutes to cure. Once cured, peel the PDMS from the template and cut it to the appropriate size. Use a puncher to create the inlet and outlet of the chip. Subsequently, employ a plasma cleaner (A.1.7) to activate the PDMS blocks with different chamber structures and the porous membrane. Seal the activated porous membrane between two PDMS blocks in a sandwich form to complete the sealing process.

**Appendix B**

(Normative Appendix)

Construction of Liver-on-a-Chip: Cell Seeding and Culture

**B.1 Instruments**

UV sterilization cabinet.

**B.2 Reagents**

B.2.1 Unless otherwise specified, all reagents used are analytical grade, and water is ultrapure water.

B.2.2 Hepatocyte culture medium: DMEM high glucose medium, supplemented with 10%～20% serum and 1% antibiotics. After preparation, store at 4°C in the refrigerator for later use.

B.2.3 Endothelial cell culture medium: ECM medium, 10%～20% serum, 1% endothelial cell growth supplement, and 1% antibiotics. After preparation, store at 4°C in the refrigerator for up to one month.

B.2.4 Cell digestion solution: Trypsin/EDTA and TrypLE.

**B.3 Procedures of Cell Seeding and Culture on Chip with Tissue-tissue Interface**

B.3.1 Chip Sterilization

Expose the chip to UV light in the UV sterilization cabinet for at least 30 minutes for sterilization.

B.3.2 Seeding of Hepatocytes

Digest the cultured hepatocytes into a single-cell suspension using cell dissociation solution (B.2.4). Prepare a specific concentration of cell suspension and seed it into the hepatocyte chamber. Allow it to incubate in the incubator for one to two days to facilitate the formation of a complete monolayer structure. Subsequently, culture the cells with hepatocyte culture medium (B.2.2).

B.3.3 Seeding of Nonparenchymal Cells

Digest endothelial cells and/or hepatic stellate cells using cell dissociation solution. Prepare a specific concentration of endothelial cell suspension or endothelial cell-hepatic stellate cell mixed cell suspension. Seed the suspension into the nonparenchymal cell chamber and allow it to stand in the incubator for 1-2 days to form a complete monolayer structure. Subsequently, culture the cells with endothelial cell culture medium (B.2.3) or mixed culture medium.

**B.4 Procedures of Cell Seeding and Culture on Chip in 3D Format**

B.4.1 Chip Sterilization

Expose the chip to UV light in the UV sterilization cabinet for at least 30 minutes for sterilization.

B.4.2 Seeding of Hepatocytes

Digest the cultured hepatocytes into a single-cell suspension using cell dissociation solution. Prepare a specific concentration of cell suspension and seed it into the microstructure array in the liver chip. Allow it to incubate in the incubator for one to two days to facilitate the formation of 3D spheroids.

**B.5 Dynamic Culture on Liver-on-a-Chip**

Once over 80% of the cells in the chip adhere to the wall or form spheres, initiate fluid flow in the culture medium using a peristaltic pump, syringe pump, or gravity flow system. Control the fluid flow rate, ensuring that the fluid shear force generated falls within the range of 0.001 dyn/cm^2^ to 5 dyn/cm^2^. Replace the culture medium daily or every other day based on the cell growth rate.

**Appendix C**

(Normative Appendix)

Construction of Liver-on-a-chip: Calculation of Shear Force

The calculation of fluid shear force involves the principles of fluid dynamics, mainly based on Newton's viscous fluid model. In a viscous fluid, the shear force (τ) can be calculated using Formula (1):

*τ* = *μ*·*du/dy*……….…..(1)

Where:

*τ* is the shear force, measured in dyn/cm^2^

*μ* is the viscosity coefficient of the fluid, measured in (dyn/cm^2^)·s.

*du/dy* is the velocity gradient, representing the rate of velocity change of the fluid perpendicular to the direction of flow, measured in s^-1^.

**Appendix D**

(Normative Appendix)

Validation of Tissue Morphology: Immunofluorescence

**D.1 Instruments**

D.1.1 Confocal Laser Scanning Microscope.

D.1.2 Inverted fluorescence microscope.

**D.2 Reagents**

D.2.1 Unless otherwise specified, all reagents used are analytical grade, and water is ultrapure water.

D.2.2 Immunofluorescence staining reagent kit.

D.2.3 H&E staining reagent kit.

D.2.4 Phosphate-buffered saline (PBS): pH 7.2~7.4.

D.2.5 Target protein antibodies.

D.2.6 Tissue Fixative: 4% paraformaldehyde.

**D.3 Validation Procedures**

D.3.1 Sample Preparation

After a specific period of cell culture in the liver chip, remove the culture medium and wash the cells with a buffer solution. Subsequently, add an appropriate volume of tissue fixative (D.2.4) to fully immerse the cells. Once the cells are completely fixed, thoroughly wash them with a buffer solution in preparation for the next experiment.

D.3.2 Immunofluorescence Staining

Perform immunofluorescence staining using the immunofluorescence staining reagent kit (D.2.2) and target protein antibodies (D.2.5) according to the instructions provided with the kit.

D.3.3 H&E Staining

Stain the cells using the H&E staining reagent kit (D.2.3) according to the instructions provided with the kit.

D.3.4 Immunofluorescence/H&E Observation

Observe and photograph the immunofluorescence or H&E staining samples using either a confocal laser scanning microscope (D.1.1) or an inverted fluorescence microscope (D.1.2).

**Appendix E**

(Normative Appendix)

Measurement of Albumin Secretion: ELISA

**E.1 Instruments**

E.1.1 Enzyme-linked immunosorbent assay (ELISA) reader.

E.1.2 Centrifuge.

**E.2 Reagents**

E.2.1 Unless otherwise specified, all reagents used are analytical grade, and water is ultrapure water.

E.2.2 ELISA detection reagent kit.

**E.3 Measurement Procedures**

E.3.1 Sample Collection

After a specific period of cell culture in the chip, collect the liver parenchymal cell culture medium within 24 hours. Centrifuge to collect the supernatant, and record the volume. Concurrently, collect the liver parenchymal cells and count the number of cells.

E.3.2 Reagent Preparation

Prepare the reagents required for ELISA detection according to the requirements, including coating solution, washing solution, blocking solution, sample dilution solution, and stop solution.

E.3.3 Drawing the Standard Curve

Dilute the standard samples in the ELISA kit (B.2.2) to a series of concentration gradients according to the kit instructions, and after measurement with the ELISA reader (B.1.1), prepare the concentration-absorbance standard curve.

E.3.4 Detection of Supernatant Sample

Dilute the culture medium supernatant appropriately according to the testing range of the ELISA kit, and perform absorbance measurement of the samples according to the ELISA kit instructions.

E.3.5 Data Processing and Calculation

Calculate the albumin content secreted by liver parenchymal cells in the chip within 24 hours according to formula (2):

*B* = *A*·*V* .................. (2)

Where:

B is the albumin content, unit: ng/24h

A is the albumin concentration, unit: ng/(mL·24h)

V is the volume of supernatant, unit: mL

Calculate the average albumin secretion of liver parenchymal cells in the chip within 24 hours according to formula (3):

*D* = *B* / *E* .................. (3)

Where:

*D* is the albumin secretion, unit: ng/(10^6^ cells·24h)

*B* is the albumin content, unit: ng/24h

*E* is the number of cells, unit: 10^6^ cells

**Appendix F**

(Normative Appendix)

Detection of Drug Metabolizing Enzyme Activity: Mass Spectrometry

**F.1 Instruments**

F.1.1 Liquid chromatography-tandem mass spectrometry (LC-MS/MS).

F.1.2 Centrifuge.

**F.2 Reagents**

F.2.1 Unless otherwise specified, all reagents used are analytical grade, and water is ultrapure water.

F.2.2 4% Bovine Serum Albumin (BSA) solution.

F.2.3 Liver cell culture medium: DMEM high glucose culture medium, supplemented with 10% serum and 1% double antibodies, prepared and stored in a refrigerator at 4°C for later use.

F.2.4 Ice-cold acetonitrile.

**F.3 Substrate Classification and Qualitative Ions in Mass Spectrometry**

Refer to **Table F.1** for substrate classification and **Table F.2** for qualitative ion pairs.

**Table F.1** Substrate classification

| CYP450 Enzyme | Substrate | Metabolite |
| --- | --- | --- |
| CYP1A2 | Phenacetin | Acetaminophen |
| CYP3A4 | Testosterone | 6β-OH-testosterone |
| CYP2B6 | Bupropion | 4-OH-bupropion |

**Note:** Substrates and metabolites are standard substances with purity greater than 98%.

**Table F.2** Qualitative Ion Pairs of Mass Spectrometry

| Substrate | Suggested Qualitative Ion Pair | Metabolite | Suggested Qualitative Ion Pair |
| --- | --- | --- | --- |
| Phenacetin | 180/110.1 | Acetaminophen | 152.1/11.1 |
| Testosterone | 289.1/97.05 | 6β-OH-testosterone | 305/269.2 |
| Bupropion | 240.1/184 | 4-OH-bupropion | 256.1/238 |

**F.4 Sample Grouping**

Refer to Table F.3 for sample grouping.

**Table F.3** Sample Grouping

| Groups | Experimental Group (n=3) | Negative Control Group (n=3) |
| --- | --- | --- |
|  | Hepatocytes |  |
| Compound Concentration | 1 μmol/L | 1 μmol/L |
| Sampling Time Points | Pre-0 h, Pre-4 h, 0 h, 0.5 h, 1 h, 2 h, and 4 h | |
| **Note 1:** All reaction systems are supplemented with 4% BSA (C.2.2) to minimize the impact of nonspecific adsorption on experimental results.  **Note 2:** Pre-0 h and Pre-4 h time points involve sampling from freshly prepared drugs and samples left in the same environment for 4 h, both considered as part of the blank control group to nullify blank control background and experimental environment influences.  **Note 3:** 0 h and 0.5 h time points: Samples are taken at intervals within 0.5 hours after adding the drug to the liver chip. | | |

**F.5 Measurement Procedures**

Prepare a substrate solution with a final concentration of 1μmol/L using the liver cell culture medium (F.2.3). Add a specific volume of the substrate solution to th`e liver parenchymal cell chamber. After thorough mixing, immediately take a certain volume of cell suspension and mix it with three times the volume of ice-cold acetonitrile (F.2.4), marking the start of the experiment at 0 hours. At the corresponding time points, take a specific volume of the cell suspension and mix it with three times the volume of ice-cold acetonitrile to terminate the reaction. After termination, centrifuge the samples and collect a certain volume of supernatant for LC-MS/MS (F.1.1) analysis.

**F.6 Calculation of Intrinsic Clearance (CLint)**

Take the natural logarithm of the ratio of peak areas obtained from the sample or positive drug as the vertical axis and time (hours) as the horizontal axis, and perform linear regression analysis (Y=a·x+b). Calculate the intrinsic clearance rate according to formula (4):

*CLint* = (0.693/*T1/2*)·(*V*/*M*) .................. (4)

Where:

*CLint* is the intrinsic clearance rate, unit: μL/(10^6^ cells·24h)

*T1/2* (h) is 0.693/K

*K* is the elimination rate constant

*V* is the incubation volume, unit: μL

*M* is the number of incubated cells, unit: 10^6^ cells.
